# Biased Random Walk Model of Neuronal Dynamics on Substrates with Periodic Geometrical Patterns

**DOI:** 10.3390/biomimetics8020267

**Published:** 2023-06-20

**Authors:** Cristian Staii

**Affiliations:** Department of Physics and Astronomy, Tufts University, Medford, MA 02155, USA; cristian.staii@tufts.edu

**Keywords:** neuron, axonal growth, neuron networks, bio-inspired neural networks, tissue engineering, stochastic processes

## Abstract

Neuronal networks are complex systems of interconnected neurons responsible for transmitting and processing information throughout the nervous system. The building blocks of neuronal networks consist of individual neurons, specialized cells that receive, process, and transmit electrical and chemical signals throughout the body. The formation of neuronal networks in the developing nervous system is a process of fundamental importance for understanding brain activity, including perception, memory, and cognition. To form networks, neuronal cells extend long processes called axons, which navigate toward other target neurons guided by both intrinsic and extrinsic factors, including genetic programming, chemical signaling, intercellular interactions, and mechanical and geometrical cues. Despite important recent advances, the basic mechanisms underlying collective neuron behavior and the formation of functional neuronal networks are not entirely understood. In this paper, we present a combined experimental and theoretical analysis of neuronal growth on surfaces with micropatterned periodic geometrical features. We demonstrate that the extension of axons on these surfaces is described by a biased random walk model, in which the surface geometry imparts a constant drift term to the axon, and the stochastic cues produce a random walk around the average growth direction. We show that the model predicts key parameters that describe axonal dynamics: diffusion (cell motility) coefficient, average growth velocity, and axonal mean squared length, and we compare these parameters with the results of experimental measurements. Our findings indicate that neuronal growth is governed by a contact-guidance mechanism, in which the axons respond to external geometrical cues by aligning their motion along the surface micropatterns. These results have a significant impact on developing novel neural network models, as well as biomimetic substrates, to stimulate nerve regeneration and repair after injury.

## 1. Introduction 

Neuronal cells are the basic building blocks of the brain and are responsible for transmitting electrical and chemical signals throughout the nervous system. The basic structure of a neuron consists of a cell body (soma), dendrites, and axons. During brain development, neurons actively grow axons that steer over distances ranging from tens to hundreds of cell diameters in length to locate target dendrites from other neurons and form neuronal networks. This process is crucial for the development and maintenance of the nervous system, and it plays an important role in learning and memory. Axonal movement is directed by the growth cone, a dynamic sensing structure found at the leading edge of the axon that carries out both sensory and locomotive functions. The cytoskeleton within the growth cone primarily consists of actin filaments arranged in lamellipodium and filopodium—like structures that extend and retract as the growth cone navigates through the surrounding extracellular environment. The lamellipodia and filopodia probe the environment for chemical, electrical, mechanical, and geometrical guidance cues and guide the axon movement in response to these extracellular cues [1,2,3,4,5,6,7].

Axonal growth is a highly complex process involving a wide range of molecular and cellular mechanisms. In the past few decades, there has been significant progress in understanding the impact of chemical cues on axonal dynamics. These include guidance of growth cones by diffusing chemical gradients such as Slits and Netrins (chemotaxis), guidance by substrate-bound biochemical cues, such as Laminin, Ephrins, and Semaphorins (haptotaxis) [1,2,3,4,5,6], as well as guidance assisted by glial or Schwann cells [7,8]. For example, it is now well-established that surface-bound chemical cues can either attract or repel growth cones [1,2,3,4,5] and that various signal transduction pathways connect the activation of growth cone sensors to modifications in the cytoskeleton dynamics [1,2,3,6,7]. Moreover, it is generally accepted that axonal elongation is largely controlled by the interplay between neuron biomechanical properties and the mechanics and geometry of the surrounding environment. Axons are composed of a complex viscoelastic network of microtubules and actin filaments which provide structural support and enable them to undergo complex mechanical deformations [3,7,9,10,11,12]. In addition, growth cones generate traction forces by pushing or pulling on the extracellular environment, while the substrate mechanics and geometry can affect the direction and speed of axonal growth [12,13].

Recent advances in microfabrication and microfluidics have enabled researchers to investigate neuronal growth in vitro, where external mechanical and geometrical cues can be controlled. For example, these studies have shown that modifying substrate stiffness has a dramatic effect on axonal elongation [4,7,13] and that periodic geometrical features patterned on the growth surface enhance axonal outgrowth and control axonal alignment [14,15,16,17,18,19,20,21,22]. The ability to guide neuronal growth in controlled environments carries significant implications for engineering novel bioinspired devices for nerve repair and neuroprosthetics applications. One of the primary objectives in tissue engineering is to create neural environments that promote axonal outgrowth and mimic in vivo physiological conditions [1,2,3,4,5,22,23,24]. Understanding the mechanisms underlying neuronal growth is, therefore, critical for our ability to direct and control neuronal growth and for developing new therapies for treating nerve injuries and nervous system disorders. Moreover, gaining an in-depth knowledge of the growth processes will allow researchers to build innovative bio-inspired neural networks that can reproduce key functional characteristics of the brain. Nevertheless, despite progress in the field, significant challenges remain regarding our fundamental understanding of the mechanisms that control neuronal growth, such as the intricate interplay between various biochemical and biophysical factors, the details of the cell–substrate interactions, the generation of traction forces, and the processes that govern neuron biomechanical responses.

In previous work, we have reported that neurons cultured on poly-D-lysine-coated polydimethylsiloxane (PDMS) substrates with periodic parallel ridge micropatterns grow axons parallel to these surface patterns [19,20,21,22]. We have demonstrated that the cell–surface interactions result in a “deterministic torque” that drives axonal alignment parallel to the surface micropatterns [20,21,22]. Our results show that axonal dynamics is governed by a closed-loop feedback control mechanism, which can be altered by the chemical treatment of the cell [21,22]. We have also measured the axonal speed and angular distributions, the diffusion (cell motility) coefficients, and the axonal bending modulus on these substrates [20,21,22]. 

## 2. Theoretical Models of Neuronal Growth 

The literature on modeling neuronal growth is extensive. Early efforts concentrated on interpreting observed growth cone movements using random walk models. For instance, Katz and colleagues [25] demonstrated that axonal elongation and retraction could be effectively described by an uncorrelated random walk. In a different report, Odde and collaborators showed that axonal extension is correlated with subsequent retraction on time scales of several minutes [26]. A related approach was employed by Buettner and colleagues [27], who extracted probabilistic rules for filopodial dynamics from time-lapse images and formalized these into a stochastic model [28]. The Goodhill group conducted significant work developing statistical models of cues binding to receptors on the growth cone [29] and employed these models to identify the constraints on sensing imposed by the gradient shape [30]. Moreover, they found that spatial sensing is more efficient than temporal sensing for a wide range of experimental cue concentrations [31]. Katz and Lasek also identified some constraints for producing ordered axonal ensembles from simple random walk models [32].

Due to the extreme complexity of the growth process’s biochemistry, it has only been possible to model the actual biophysical mechanisms in a few special cases. For example, Segev and Ben-Jacob modeled the self-wiring of a neural network in the presence of diffusive factors [33]. They employed graph-theoretic tools, such as counting neighbors, to characterize the networks formed and to compare the model predictions with experimental observations. Van Ooyen’s group [34] developed simulations of multiple axons’ dynamics in complex domains with multiple guidance factors. Mogilner and Rubenstein [35] formulated a detailed mechanical model of filopodia to determine the optimal length. Padmanabhan and Goodhill incorporated a molecular feedback loop mechanism on pathways crucial for cytoskeletal control in the growth cone [36]. This model generates unimodal or bistable growth states for axons, depending on the rates of point contact assembly. When combined with a stochastic model that provides angular distributions, the model could be utilized in a random walk with rest periods, where bouts of growth and rest are based on the changing results of this bistable switch mechanism as the growth cone interacts with its environment [36]. In recent work, Lin and collaborators constructed models of axons consisting of a small number of separate compartments and attempted to predict the growth cone’s response to external chemical gradients [37].

Another common approach employs Langevin and/or Fokker–Planck equations to model the stochastic processes underlying neuronal growth and to describe the collective behavior of ensembles of axons within the network. Stochastic processes arise from a variety of sources, including fluctuations in the signaling molecules detected by the growth cone, polymerization of actin filaments, formation of lamellipodia and filopodia, and intercellular interactions. By explicitly deriving probability distributions for the ensemble of axons as a solution to a Fokker–Planck equation, it is possible to generate predictions about the formation of neuronal networks under different conditions and to test different growth mechanisms from experimentally observed results [38]. For instance, Hentschel and van Ooyen [39] demonstrated that a combination of chemoattractant and chemorepellent factors could account for the bundling, guidance, and subsequent de-bundling of axons towards specific target regions. Maskery and Shinbrot [40] used Langevin simulations to predict minimum detectable chemical gradients. Pearson and colleagues [41] solved the Fokker–Planck equation to describe the path of the growth cone in the absence of external chemical cues. Goodhill and collaborators [42] developed a detailed growth model incorporating filopodia extension and retraction and ligand binding to simulate axonal trajectories in the presence of external chemical gradients. Betz and collaborators [43] employed Fokker–Planck to quantify the stochastic fluctuations of the growth cone lamellipodia and demonstrated that the observed bimodal behavior of the growth cone emerges from the internal actin polymerization processes.

In our previous work, we demonstrated that Langevin and Fokker–Planck equations yield a general framework for predicting growth cone dynamics and describing the influence of various environmental cues on neuronal growth [16,17,18,19,20,21,22]. For example, our early results showed that the growth dynamics of neuronal cells cultured on glass substrates coated with poly-D-lysine (PDL) is governed by a linear Langevin equation with stochastic white noise, resulting in a regulatory mechanism for axonal growth rates on these surfaces [18]. We have also employed Langevin and Fokker–Planck equations to quantify axonal growth and measure the growth cone diffusion coefficient for cells grown on surfaces with ratchet-like topography consisting of asymmetric tilted nanorods [16,17]. In a series of recent publications, we have demonstrated that periodic geometrical micropatterns impart strong directional bias to axonal growth [19,20,21,22]. We have shown that the dynamics of the growth cones on these surfaces can be described by considering the competition between stochastic events and deterministic factors based on geometrical and mechanical guidance cues. Our results show that axonal growth is controlled by a feedback mechanism in which the growth cone detects external geometrical and mechanical cues and continuously adjusts its trajectory in response to these environmental features. 

In this paper, we combine experimental observations with theoretical analysis to develop a detailed stochastic model of axonal growth on surfaces with periodic micropatterns. We demonstrate that the periodic geometrical features generate a constant drift term applied to the growth cone, and the stochastic components produce a random walk motion along the axonal growth direction. We use this model to calculate the average axonal velocity, mean squared velocity, and mean squared length and show that the model predictions are in excellent agreement with experimental data. Our results have important implications for the fundamental understanding of neuronal growth and the formation of neuronal networks, as well as for developing novel bio-inspired neural networks and for advanced bioengineering substrates to facilitate nerve repair and regeneration. 

## 3. Materials and Methods

In this study, we used cortical neurons sourced from embryonic day 18 rats. The brain tissue protocol was approved by the Tufts University Institutional Animal Care Use Committee and complied with the NIH Guide for the Care and Use of Laboratory Animals. For cell dissociation and culture, we employed established protocols referenced in our previous reports [9,10,11,16,17,18,19,20,21,22]. Previous immunostaining experiments conducted by our group have verified high neuronal cell purity in these cultures [9]. The neuronal cells were plated on micropatterned polydimethylsiloxane (PDMS) substrates coated with poly-D-lysine (PDL) at a surface density of 4000 cells/cm^2^. As indicated in our earlier studies, neurons cultured at comparatively low densities (between 3000 and 7000 cells/cm^2^) develop long axons that are optimal for investigating growth dynamics on surfaces with various external cues [16,17,18,19,20,21,22]. As the cell density increases, the degree of axonal alignment decreases, which reflects the fact that the axons are branching more often and are making more connections at higher densities, therefore deviating from the direction imparted by the surface geometry. This implies that high surface densities (higher than 8000 cells/cm^2^) where neuron–neuron signaling is important are also sub-optimal for exploring the effect of geometrical cues on neuronal growth. The cell density of 4000 cells/cm^2^ chosen in this paper is in the middle of this optimal density range. We have also demonstrated that, in contrast to axons, dendrites do not exhibit significant growth alignment along the directions of the micropatterns [22]. Experimental data also show that the formation of axon bundles (fasciculation) is a relatively rare process in our experiments (less than 10%). Based on these experimental details, in this paper, we take the neuron–neuron signaling interactions to be negligible compared to cell–substrate interactions.

**Figure 1:**
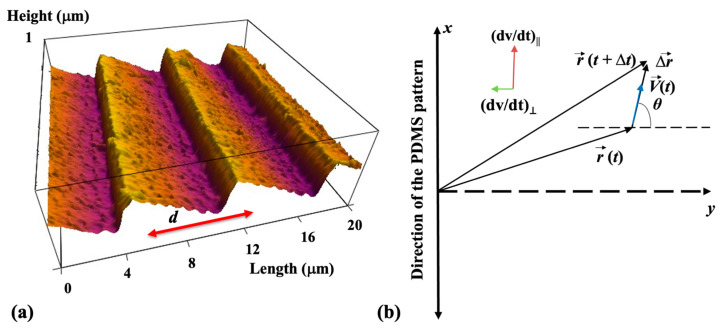
(**a**) Atomic force microscope (AFM) topographic image of a PDL-coated PDMS micropatterned surface. The image shows that the micropatterns are periodic in the *y* direction with the spatial period *d =* 7 μm and have a constant depth of approximately 0.5 μm. (**b**) Schematic of the coordinate system. The *x*-axis is defined as the axis parallel to the direction of the PDMS patterns. The directions of the parallel and perpendicular components of the acceleration are shown in the figure inset.

The periodic micropatterns on PDMS surfaces consist of parallel ridges separated by troughs. To prepare these patterns, we used a simple fabrication method based on imprinting diffraction grids onto PDMS substrates (details about the microfabrication methods are provided in the Supplementary Materials). The distance between two neighboring ridges on these surfaces is defined as the pattern spatial period *d.* An example of an atomic force microscope (AFM) image of the micropatterns is shown in Figure 1a. For the results discussed in this paper, we use surfaces with *d = 7* μm (Figure 1a). 

The micropatterned surfaces were spin-coated with PDL (Sigma–Aldrich, St. Louis, MO, USA) solution with 0.1 mg/mL concentration. Growth surfaces were imaged using an MFP3D atomic force microscope (AFM) equipped with a BioHeater closed fluid cell and an inverted Nikon Eclipse Ti optical microscope (Micro Video Instruments, Avon, MA, USA). Growing neuronal cells have been imaged using fluorescence microscopy. Fluorescence images have been acquired using a standard fluorescein isothiocyanate-FITC filter; excitation: 495 nm and emission: 521 nm (details provided in the Supplementary Materials).

Data analysis. Growth cone position and axonal length have been tracked and quantified using ImageJ version 1.53h 04, National Institute of Health, Bethesda, MS, USA). The displacement of the growth cone was measured by tracing the change in the center of the growth cone position. To measure the growth cone velocities, the neurons were imaged using fluorescence microscopy every Δ*t* = 5 min for a total period of 30 min for images taken at *t_culture_* = 10, 15, 20, 25, 30, 35, 40, 45, and 50 h after cell culture. The 5 min time interval between measurements was selected such that the displacement $\Delta \vec{r}$ of the growth cone in this interval satisfies two conditions: (1) its magnitude is greater than the experimental precision of our measurement (~0.1 μm) [19,20,21,22]; (2) the ratio $\Delta \vec{r}/\Delta t$ approximates the instantaneous velocity $\vec{V}$ of the growth cone. The growth angle *θ* is measured with respect to the *y*-axis (the growth angle and the *x* and *y* axes are defined in Figure 1b). To obtain the velocity distributions, the range of growth cone velocities at each time point was binned into intervals of equal size. Experimentally, the velocity correlation function for growth in the *x* direction (see below) is obtained with the formula [19,20]:

(1) $$\langle V\left(t_{1}\right)\cdot V(t_{2})\rangle =\frac{1}{N}\cdot \overset{N}{\underset{i=1}{\sum }}\left[V_{i}\left(t_{1}\right)\cdot V_{i}\left(t_{2}\right)\right]$$

where *N* is the total number of growth cones and $V_{i}\left(t_{1}\right),\;\;V_{i}\left(t_{2}\right)$ represent the *x* components of the velocity for the *i*th growth cone at times *t*_1_ and *t*_2_, respectively.

## 4. Results 

### 4.1. Neuronal Growth on PDMS Substrates

**Figure 2:**
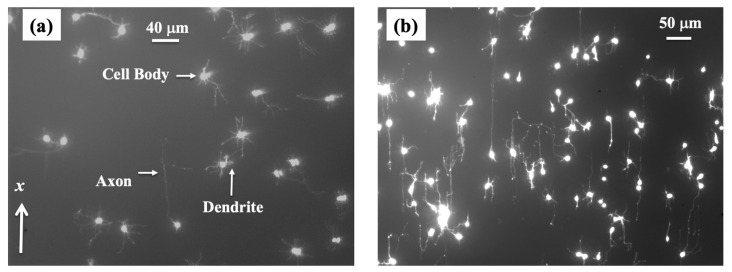
Fluorescence (Tubulin Tracker Green) images showing examples of axonal growth for cortical neurons cultured on PDL-coated PDMS surfaces with periodic micropatterns. (**a**) Example of growth image for neurons, acquired at *t_culture_* = 10 h after plating. The direction of the micropatterns is shown by the *x*-axis. The figure also shows the main structural components of a neuronal cell. Cortical neurons typically grow a long process (axon) and several shorter processes (dendrites). The axons are identified by their morphology, and the growth cone is located at the tip of the axon. (**b**) Example of growth for neurons imaged at *t_culture_* = 50 h after plating. The image shows a high degree of axonal alignment along the direction *x* of the micropatterns.

Cortical neurons are cultured on PDL-coated PDMS surfaces with periodic micropatterns with the spatial pattern period *d* = 7 μm (Figure 1a). Axonal growth on these surfaces is quantified at different time points after cell plating: *t_culture_* = 10, 15, 20, 25, 30, 35, 40, 45, and 50 h. The reason for choosing these growth conditions is explained below. Figure 2 shows examples of images for axonal growth on these substrates taken at *t_culture_* = 10 h (Figure 1a) and *t_culture_* = 50 h (Figure 2b). 

We have previously demonstrated that the axons of neurons cultured on micropatterned PDMS surfaces tend to grow along the directions of the surface patterns and that the degree of axonal alignment increases with time [19,20,21,22]. Furthermore, axons display maximum alignment along PDMS patterns for surfaces with the pattern spatial period *d* matching the linear dimension of the growth cone, that is, when *d* is of the order of a few micrometers [20,21]. Our work also shows that axons tend to grow on top of the periodic ridges, with typically a single axon extending along any single PDMS micropattern ridge [14,19,22]. The experimental data shown in Figure 2 is in agreement with our previous findings. In reference [20], we have demonstrated that the axonal growth on these surfaces is governed by two non-linear Langevin equations of motion for the velocity *V*:

(2) $${\left(\frac{dV}{dt}\right)}_{\left|\right|}=a_{0}|\sin\theta |-\gamma_{1}\cdot V-\gamma_{2}\cdot V^{2}+\Gamma \left(t\right)$$

(3) $${\left(\frac{dV}{dt}\right)}_{⊥}=a_{1}\cos\theta +\Gamma_{⊥}\left(t\right)$$

In the above equations, ${\left(dV/dt\right)}_{\left|\right|}$ and ${\left(dV/dt\right)}_{⊥}$ are, respectively, the parallel and perpendicular components of the growth cone acceleration, *θ* represents the growth angle (these terms are defined in Figure 1b), and *a*_0_, *a*_1_, *γ*_1_, *γ*_2_ are velocity-independent parameters that depend on the pattern spatial period *d*. We have shown that all these parameters can be experimentally measured by analyzing the spatial and temporal evolutions of axonal growth [19,20,21,22]. In the above equations Γ and $\Gamma_{⊥}$ represent the stochastic contributions for parallel and perpendicular growth, satisfying the conditions for Gaussian white noise with zero mean, characteristic of uncorrelated Wiener processes [20]:

(4) 〈Γ(t)〉=0

(5) $$\langle \Gamma \left(t_{1}\right)\Gamma (t_{2})\rangle =\sigma^{2}\cdot \delta \left(t_{1}-t_{2}\right)$$

with similar expressions for $\Gamma_{⊥}$. In Equations (4) and (5) σ is a term that quantifies the strength of the noise (the variance of the stochastic distribution) and $\delta \left(t_{1}-t_{2}\right)$ is the Dirac delta function. 

We now introduce two key experimental observations that allow us to simplify the non-linear equations of motions (2) and (3). First, as we have noted above, the axonal alignment along the pattern is increasing with time. The perpendicular component of the acceleration (Equation (3)) acts as a deterministic torque that tends to align the growth cone motion along the direction *x* of the pattern. The experimental data show that the magnitude of the perpendicular acceleration has maximum values at the beginning of the growth when many axons elongate in directions perpendicular to the micropattern. This process corresponds to the maximum ${\left(dV/dt\right)}_{⊥}$ for θ≈0, as predicted by Equation (3). As time increases, more and more axons align with the micropatterns and continue their growth along the *x* direction, characterized by $\theta \approx \frac{\pi }{2}$ (or $\theta \approx \frac{3\pi }{2}$). In this case, the deterministic torque in Equation (3) is negligible: $a_{1}\cos\theta \;\approx 0$, and the axonal motion becomes quasi-one-dimensional along the *x* direction. The stochastic contributions produce fluctuations along this average direction of motion. Experimentally, these conditions are satisfied for growth times *t_culture_* in the interval 10–50 h. For *t_culture_* > 50 h most axons start to form connections with other neurons, and the growth process stops. For the rest of the paper, we define the *observation time t* as: *t* = *t_culture_* – 10 h and measure axonal growth in time increments of 5 h in the interval: 0≤t≤40 h (corresponding to the interval 10–50 h after plating). 

The second observation is that the parameter *γ*_1_ in Equation (2) is approximately constant $\gamma_{1}\;\approx 0.1\;{\mathrm{hr}}^{-1},$ whereas the parameter *γ*_2_ decreases with increasing spatial period *d*, as we have demonstrated in our previous work [20]. In particular, for *d* = 7 μm, we have reported that $\gamma_{2}\;\approx 10^{-3}\;{\mu m}^{-1},$ [20], which implies that for growth velocities ~10 μm/hr, we have that:

(6) $$\gamma_{2}\cdot V^{2}\ll \gamma_{1}\cdot V$$

**Figure 3:**
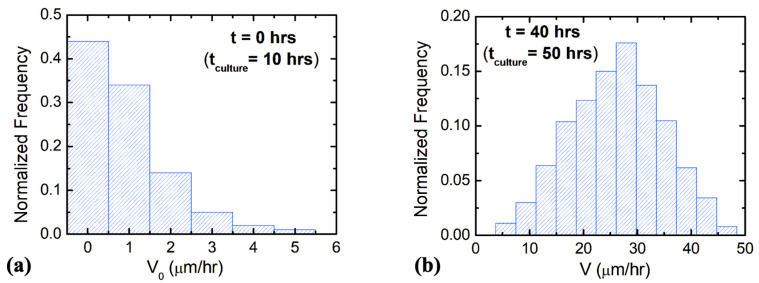
Examples of normalized distributions for the parallel component of the growth cone velocity measured on micropatterned PDMS surfaces with pattern spatial period *d =* 7 μm. (**a**) Velocity distribution for N = 85 different growth cones measured at *t* = 0 h (10 h after plating, see main text). (**b**) Velocity distribution for N = 172 different growth cones measured at *t* = 40 (50 h after plating).

This condition holds for the observed axonal growth on surfaces with d ≥7 μm [20]. Figure 3 shows examples of normalized distributions for the parallel component (along the *x*-axis) of the growth cone velocity measured at *t =* 0 h (Figure 3a) and *t =* 40 h (Figure 3b), respectively. The experimental data show the measured velocities are of the order of a few tens μm/hr such that: $V\ll \gamma_{1}/\gamma_{2}\approx 100$ μm/hr.

Summarizing the above experimental observations, we conclude that in the time interval 0≤t≤40 h, the axonal growth is one-dimensional along the direction of the micropatterns (*x*-axis in Figure 2a), and it is described by the following stochastic equation for the velocity *V* of the growth cones:

(7) $$\left(\frac{dV}{dt}\right)=a_{0}-\gamma_{1}V+\Gamma \left(t\right)$$

which is obtained from Equation (2) by employing Equation (6) and the condition $\theta \approx \frac{\pi }{2}$. We take Equation (7) as the starting point for the analysis presented in the rest of this paper.

### 4.2. Axonal Dynamics along Parallel Micropatterns 

Having established the conditions for the quasi-one-dimensional growth of axons on micropatterned PDMS substrates, we now proceed to analyze the predictions of Equation (7) and compare these predictions with the experimental results. Integrating Equation (7) gives:

(8) $$V(t)=\frac{a_{0}}{\gamma_{1}}+(v(0)-\frac{a_{0}}{\gamma_{1}})\cdot e^{-\gamma_{1}t}+\int_{0}^{t}e^{-\gamma_{1}(t-q)}\Gamma \left(q\right)dq$$

where v(0) is the initial growth cone velocity. Next, we use Ornstein–Uhlenbeck’s method [44] to calculate average values over several independent realizations of the stochastic term Γ (henceforth, the symbol 〈X〉 will denote the average value for the quantity *X*). Using Equations (4) and (8), we obtain the average value of the axonal velocity 〈V(t)〉:

(9) $$\langle V(t)\rangle =\left(V_{0}-\frac{a_{0}}{\gamma_{1}}\right)\cdot e^{-\gamma_{1}t}+\frac{a_{0}}{\gamma_{1}}$$

where $\langle V_{0}=v\left(0\right)\rangle$. The velocity correlation function at two arbitrary growth times *t*_1_ and *t*_2_ (with *t*_1_ > *t*_2_) can be computed from Equations (8) and (9):

(10) $$\begin{array}{ll} \langle V\left(t_{1}\right)\cdot V\left(t_{2}\right)\rangle & \\ & ={\left(\frac{a_{0}}{\gamma_{1}}\right)}^{2}+{\left(V_{0}-\frac{a_{0}}{\gamma_{1}}\right)}^{2}\cdot e^{-\gamma_{1}\left(t_{1}+t_{2}\right)}+\frac{a_{0}}{\gamma_{1}}\left(V_{0}-\frac{a_{0}}{\gamma_{1}}\right)\cdot \left(e^{-\gamma_{1}t_{1}}+e^{-\gamma_{1}t_{2}}\right)\\ & +\int_{0}^{t_{1}}dq_{1}\int_{0}^{t_{2}}dq_{2}e^{-\gamma_{1}\left(t_{1}-q_{1}\right)}\cdot e^{-\gamma_{1}\left(t_{2}-q_{2}\right)}\cdot \langle \Gamma \left(q_{1}\right)\Gamma \left(q_{2}\right)\rangle \\ & ={\left(\frac{a_{0}}{\gamma_{1}}\right)}^{2}+{\left(V_{0}-\frac{a_{0}}{\gamma_{1}}\right)}^{2}\cdot e^{-\gamma_{1}\left(t_{1}+t_{2}\right)}+\frac{a_{0}}{\gamma_{1}}\left(V_{0}-\frac{a_{0}}{\gamma_{1}}\right)\cdot \left(e^{-\gamma_{1}t_{1}}+e^{-\gamma_{1}t_{2}}\right)\\ & +\frac{\sigma^{2}}{2\gamma_{1}}\cdot \left(e^{-\gamma_{1}\left(t_{1}-t_{2}\right)}-e^{-\gamma_{1}\left(t_{1}+t_{2}\right)}\right) \end{array}$$

where we have used Equation (5) to obtain the equality in the last line of Equation (10). In particular, setting *t*_1_ = *t*_2_ = *t* in Equation (10), we get the mean squared velocity for axonal growth as a function of time:

(11) $$\langle V^{2}\left(t\right)\rangle ={\left(\frac{a_{0}}{\gamma_{1}}\right)}^{2}+{\left(V_{0}-\frac{a_{0}}{\gamma_{1}}\right)}^{2}\cdot e^{-2\gamma_{1}t}+\frac{2a_{0}}{\gamma_{1}}\left(V_{0}-\frac{a_{0}}{\gamma_{1}}\right)\cdot e^{-\gamma_{1}t}+\frac{\sigma^{2}}{2\gamma_{1}}\cdot \left(1-e^{-2\gamma_{1}t}\right)$$

**Figure 4:**
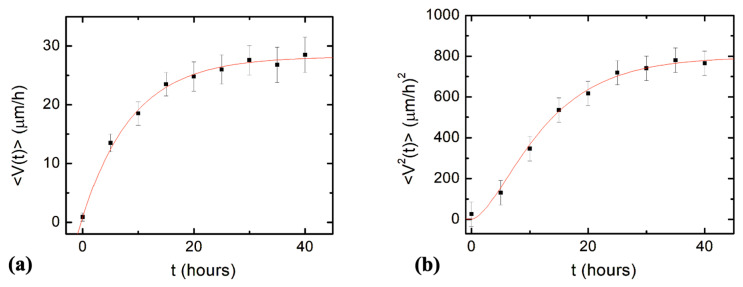
(**a**) Variation of the average axonal velocity along the direction of the micropattern (*x* direction in Figure 1) with time. The data points represent the experimentally measured average velocity at different times. The continuous red curve is fit to the data with Equation (9). (**b**) Variation of the axonal mean squared velocity along the direction of the micropattern with time. The data points represent the experimentally measured mean squared velocity at different times. The continuous red curve is fit to the data with Equation (11). Each data point in (**a**) and (**b**) was obtained by measuring between N = 64 and N = 182 axons. Error bars indicate the standard error of the mean. The fit of the data points with Equations (9) and (11) gives the constant drift coefficient *a*_0_, damping coefficient *γ*_1_, and the variance $\sigma^{2}$ of the stochastic term Γ (see text).

Next, we compare the predictions of the theoretical model given by Equations (7)–(11) with experimental data measured for axonal growth along the direction of the micropattern. Figure 4a shows the experimental data for the average axonal velocity vs. time, together with the fit to the data points with Equation (9). Figure 4b shows the experimental data for the mean squared velocity vs. time, together with the fit to the data points with Equation (11). 

The average initial velocity for growth cones is found from the velocity distribution at *t* = 0 h (Figure 3a): *V*_0_ = 0.9 μm/hr. From the fit of the data in Figure 4a,b with Equations (9) and (11), respectively, we obtain the values for the constant drift coefficient: $a_{0}=\left(3.1\;\pm 0.5\right)\;\mu m/\mathrm{hr},$ the variance for the stochastic noise: $\sigma^{2}=(0.52\;\pm 0.08){\;\mu m}^{2}/{\mathrm{hr}}^{3},$ and damping coefficient for axonal growth: $\gamma_{1}=(0.11\;\pm 0.04){\;\mathrm{hr}}^{-1}$.

By integrating Equation (8), we can also calculate the position of the growth cone as it moves along the *x*-axis:

(12) $$x\left(t\right)=\int_{0}^{t}V\left(q\right)dq=\frac{a_{0}}{\gamma_{1}}\cdot t+\left(\frac{v\left(0\right)}{\gamma_{1}}-\frac{a_{0}}{\gamma_{1}^{2}}\right)\cdot \left(1-e^{-\gamma_{1}t}\right)+\int_{0}^{t}dqe^{-\gamma_{1}q}\int_{0}^{q}e^{\gamma_{1}s}\Gamma \left(s\right)ds$$

The axonal mean squared length $\langle L^{2}\left(t\right)\rangle$ as a function of time is given by:

(13) $$\begin{array}{c} \langle L^{2}\left(t\right)\rangle =\langle {\left(x\left(t\right)-x_{0}\right)}^{2}\rangle =\langle {\left[\int_{0}^{t}V\left(t_{1}\right)dt_{1}\right]}^{2}\rangle =\langle \int_{0}^{t}V\left(t_{1}\right)dt_{1}\cdot \int_{0}^{t}V\left(t_{2}\right)dt_{2}\rangle \\ =\int_{0}^{t}dt_{1}\int_{0}^{t}\langle V\left(t_{1}\right)\cdot V\left(t_{2}\right)\rangle dt_{2} \end{array}$$

By using Equation (10) and the Gaussian white noise conditions in Equations (4) and (5), we get:

$$\begin{array}{r} \langle L^{2}\left(t\right)\rangle =\frac{\sigma^{2}}{2\gamma_{1}}\cdot \left[\int_{0}^{t}dt_{1}\int_{0}^{t}dt_{2}e^{-\gamma_{1}\left(t_{1}-t_{2}\right)}-{\left(\int_{0}^{t}dt_{1}e^{-\gamma_{1}t_{1}}\right)}^{2}\right]+{\left(V_{0}-\frac{a_{0}}{\gamma_{1}}\right)}^{2}\cdot {\left(\int_{0}^{t}dt_{1}e^{-\gamma_{1}t_{1}}\right)}^{2}\\ =\frac{\sigma^{2}}{2\gamma_{1}}\cdot \left[2\left(\frac{t}{\gamma_{1}}+\frac{e^{-\gamma_{1}t}-1}{\gamma_{1}{}^{2}}\right)-\frac{{\left(e^{-\gamma_{1}t}-1\right)}^{2}}{\gamma_{1}{}^{2}}\right]+{\left(V_{0}-\frac{a_{0}}{\gamma_{1}}\right)}^{2}\cdot \frac{{\left(e^{-\gamma_{1}t}-1\right)}^{2}}{\gamma_{1}{}^{2}}\;\;\;\; \end{array}$$

which simplifies to:

(14) $$\langle L^{2}\left(t\right)\rangle =\frac{\sigma^{2}}{\gamma_{1}^{2}}\cdot t+\frac{\sigma^{2}}{2\gamma_{1}^{3}}\cdot \;\left(4e^{-\gamma_{1}t}-e^{-2\gamma_{1}t}-3\right)+{\left(V_{0}-\frac{a_{0}}{\gamma_{1}}\right)}^{2}\cdot \frac{{\left(e^{-\gamma_{1}t}-1\right)}^{2}}{\gamma_{1}{}^{2}}$$

**Figure 5:**
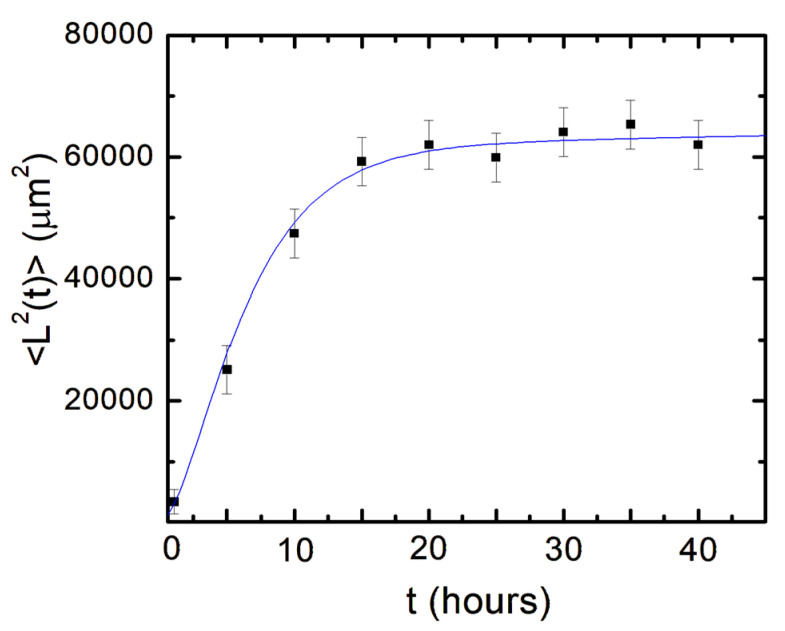
Variation of the axonal mean squared length with time. Data points represent experimentally measured axonal mean square length at different times. Error bars indicate the standard error of the mean for each data set. The blue curve is the plot of Equation (14) without any additional free parameters.

In Figure 5, we show the experimental data for the axonal mean squared length (black data points) as well as the plot of Equation (14) (blue curve) without the introduction of any additional free parameters (all parameters appearing in Equation (14) have been measured from the data fit in Figure 4). 

Equation (14) shows that for large time scales: $\gamma_{1}\cdot t\gg 1$ the mean squared length reduces to a constant drift term plus a term proportional to the time *t*:

(15) $$\langle L^{2}\left(t\right)\rangle \approx \frac{1}{\gamma_{1}{}^{2}}{\left(V_{0}-\frac{a_{0}}{\gamma_{1}}\right)}^{2}+\frac{\sigma^{2}}{\gamma_{1}^{2}}\cdot t$$

The second term in Equation (15) is characteristic of a diffusive process in which the axonal mean squared length increases linearly with time. In this regime, we can define a diffusion coefficient for the growth cones by analogy with ordinary Brownian motion [44]:

(16) $$D=\int_{0}^{\infty }\langle V\left(0\right)\cdot V\left(t\right)\rangle dt$$

Using Equation (10) with the conditions: $t_{1}=t,\;\;t_{2}=0$ and $\gamma_{1}\cdot t\gg 1$, and subtracting the constant drift term, we get the diffusion coefficient:

(17) $$D=\int_{0}^{\infty }\langle V\left(0\right)\cdot V\left(t\right)\rangle dt\;\approx \int_{0}^{\infty }\frac{\sigma^{2}}{2\gamma_{1}}e^{-\gamma_{1}t}dt=\frac{\sigma^{2}}{2\gamma_{1}^{2}}$$

By plugging in the measured values for the parameters $\sigma^{2}\;\approx 0.52\;{\;\mu m}^{2}/{\mathrm{hr}}^{3},$ and $\gamma_{1}\approx 0.11\;{\;\mathrm{hr}}^{-1}$ we obtain $D\approx 21\;{\mu m}^{2}/\mathrm{hr}$.

Finally, by employing Equation (17) for the diffusion coefficient, we can rewrite the expression for the axonal mean squared length (Equation (14)) to obtain:

(18) $$\langle L^{2}\left(t\right)\rangle =2Dt+\frac{D}{\gamma_{1}}\cdot \;\left(4e^{-\gamma_{1}t}-e^{-2\gamma_{1}t}-3\right)+{\left(V_{0}-\frac{a_{0}}{\gamma_{1}}\right)}^{2}\cdot \frac{{\left(e^{-\gamma_{1}t}-1\right)}^{2}}{\gamma_{1}{}^{2}}$$

This equation shows that the axonal dynamics on the micropatterned PDMS substrates are characterized by a biased random walk, in which the surface geometry imparts a constant drift term to the growth cone, and the stochastic components lead to a diffusive motion around the average growth direction. 

## 5. Discussion

Neuronal growth is the result of the complex interactions between deterministic cues and stochastic factors that affect the growth cone. Deterministic influences include substrate geometry and mechanics, as well as external electric fields and chemical gradients. Stochastic components originate in processes such as the polymerization of actin filaments, random fluctuations in the intercellular signaling, and the low concentration of the chemoattractant and chemorepellent biomolecules [1,2,3,4,5,6,7]. In this paper, we show that parallel geometrical patterns promote axonal alignment along the direction of the patterns. Thus, surface geometry represents the primary deterministic factor directing neuronal growth on micropatterned PDMS surfaces. The inherent stochastic nature of neuronal growth is characterized by Gaussian white noise. In the previous sections, we have demonstrated that axonal dynamics on these substrates are described by a biased random walk model given by Equations (7)–(18). This model shows that the overall movement of the growth cone has two components: (1) a uniform drift along the direction of the PDMS micropatterns (defined as the *x*-axis in Figure 1 and Figure 2), a random Brownian-like motion around these main growth directions. The parameters that characterize this motion are the drift coefficient *a*_0_, the damping coefficient *γ*_1_, and the strength σ of the stochastic noise. We use this theoretical model to fit the experimental data for the time dependence of the axonal average and mean squared velocities (fits to the data are represented by the continuous red curves in Figure 4). From the data fit, we obtain the values of the parameters for axonal growth: $a_{0}=\left(3.1\;\pm 0.5\right)\;\mu m/\mathrm{hr},\;\;\sigma^{2}=(0.52\;\pm \;0.08){\;\mu m}^{2}/{\mathrm{hr}}^{3},$ and $\gamma_{1}=(0.11\;\pm \;0.04){\;\mathrm{hr}}^{-1}$. Figure 5 shows excellent agreement between the experimental measurements for the axonal mean squared length and the theoretical prediction of the drift-diffusion model, given by Equation (14), which is plotted without any additional adjustable parameters (blue curve in Figure 5).

The drift-diffusion regime is characterized by an increase in the axonal mean squared velocity along the direction of the pattern, with a characteristic time $\tau =1/\gamma_{1}\approx$ 9 h (or ~19 h after neuron plating), and a cell motility (diffusion) coefficient $D\approx 21\;{\mu m}^{2}/\mathrm{hr}$. This value is close to the diffusion coefficients we have measured in previous work for neuronal growth on several types of two-dimensional substrates [17,18,19,20,21,22] and is comparable to diffusion coefficients reported in the literature for other types of cells [45,46,47]. We note that the diffusion coefficient is proportional to the variance of the stochastic term (Equation (17)), and therefore it represents a measure of the axonal random walk superimposed to the overall drift. Indeed, from Equation (18), we have that for large *t* (that is for $t\gg 1/\gamma_{1}\approx$ 9 h), the axonal mean squared length is given by:

(19) $$\langle L^{2}\left(t\right)\rangle \approx 2Dt+\frac{1}{\gamma_{1}{}^{2}}{\left(V_{0}-\frac{a_{0}}{\gamma_{1}}\right)}^{2}$$

that is a sum between a diffusive and a constant drift term. However, Equations (16)–(19) have a different interpretation than the corresponding relations that characterize a simple Brownian motion [44]. In particular, let us assume that the fluctuation–dissipation theorem for a system at thermodynamic equilibrium holds for axonal growth. Then applying Einstein’s relation for diffusion [44]: $D=k_{B}\cdot T/\gamma_{1}$ (where *k_B_* and *T* are the Boltzmann constant and temperature, respectively) we obtain a fictitious temperature for growth of the order of 10,000 K. Clearly, neuronal growth and, more generally, cellular motility are not thermal equilibrium processes. Nevertheless, in this paper, we refer to *D* as the cellular diffusion coefficient, following the conventional terminology found in the literature [22,26,27,28,29,30,31,32,33,34,45,46,47]. 

We emphasize that these results are valid for intermediate growth times *t_culture_* in the interval 10–50 h after neuron culture. For our analysis, we have defined the observation time *t* as: *t* = *t_culture_* – 10 h and measured growth in time increments of 5 h in the interval: 0≤t≤40 h. At earlier times, the growth cone had a two-dimensional movement on the surface which is described by Equations (2) and (3). As time progresses, the axon is steered toward the surface micropattern (i.e., along the directions characterized by $\theta =\frac{\pi }{2}$, $\frac{3\pi }{2}$, see Figure 1 and Figure 2) by the deterministic torque in Equation (3). Thus, for growth times longer than 10 h after plating, the movement of the growth cone is well approximated by one-dimensional motion along the direction of the micropatterns with stochastic fluctuations along this direction. In our previous work, we investigated the dependence of neuronal growth on the spatial periodicity *d* of the geometrical patterns [20,21,22]. Our previous results show that axons display a maximum degree of alignment when *d* is in the range 3–5 μm, that is, when *d* is close to the linear dimension of the average growth cone [20,22]. The spatial period of the geometrical patterns in the current study is *d* = 7 μm (Figure 1), which is larger than the typical values for the size of the growth cone. The experimental data show that, in this case, the degree of axonal alignment and the average growth velocity are smaller. This leads to a simplification in the equations of motion; that is, Equations (2) and (3) are replaced by Equation (7), as we have discussed in the previous sections. 

These results support our earlier observations that axons navigate and follow geometrical patterns via a contact—guidance mechanism [20,21,22]. Contact guidance refers to the phenomenon by which cells respond to mechanical and geometrical cues in their surrounding environment to direct their movement and growth. This phenomenon has been observed for several types of cells, such as neurons, fibroblasts, and tumor cells [48,49,50,51]. In addition to its role in cell migration, contact guidance has been shown to affect cell proliferation, differentiation, and even gene expression [51,52,53]. The biophysical mechanisms underlying contact guidance are not yet fully understood. However, previous work has shown that in contact guidance, the intracellular actin filaments interact with the extracellular matrix (ECM) on the surrounding substrate and that these interactions are facilitated by a particular type of proteins known as integrins [50,51,52,53]. These molecules control the cell–substrate forces by attaching actin filaments to ECM and developing special cell attachment sites called focal adhesion points. These interactions cause cells to sense and respond to substrate features such as grooves, ridges, or pores in both in vivo and in vitro experiments [3,4,7,24,48,49,50,51]. For example, prior studies have also shown that contact guidance involves the activation of signaling pathways (such as Rho-associated proteins) that play an important role in regulating the dynamics of actin filaments and the formation of focal adhesion points [52]. 

In the case of neuronal growth, the contact guidance mechanism presents several unique characteristics. The interaction between the growth cone and the substrate results in the formation of a “molecular clutch”. This “clutch” anchors the cytoskeleton to the substrate, thereby enabling the growth cone to extend the lamellipodia and filopodia [1,2,3,48]. The actin filaments push against the substrate through the clutch, thus generating traction forces that contribute to the advancement of the growth cone. The process is regulated by the contractility of the motor protein myosin II. In previous work, we have reported the importance of the cytoskeleton and molecular motors (myosin II) in controlling the axonal alignment on directional substrates [14,21,22]. Myosin II controls the dynamics of actin filaments at the leading edge of the growth cone and thus plays an essential role in the generation of external forces and the maturation of curvature-sensing proteins and focal adhesion points. In our work, we have treated neurons with Blebbistatin and/or Y-27,632, two chemical compounds that inhibit the activity of myosin II and thus alter the dynamics of actin filaments [14,21,22]. Our results show that inhibition of cytoskeletal dynamics via chemical treatment results in a decrease in axonal alignment and, thus, in the tendency of growth cones to follow geometrical patterns. We have demonstrated that the chemical treatment of neurons affects the generation of traction forces and the development of focal adhesion points and ultimately leads to a decreased degree of alignment of the axons with the surface patterns. Similar alteration of cytoskeletal dynamics also seems to take place in many neurogenerative diseases or following neuron injury [1,2,3,4,50]. However, the alteration of the regulatory mechanisms and their interaction with molecular motors and cellular cytoskeleton in the disease states are not completely understood [48,49,50]. 

We have also performed combined traction force—atomic force microscopy measurements and demonstrated that the clutch mechanism leads to an increase in cell–substrate traction forces, as well as to an overall enhancement of neuron elastic modulus during axonal extension [12]. Our previous results also show that the growth cone wraps around the high curvature features of the PDMS micropatterns, thereby resulting in an increase in the density of focal adhesion points when the axons are aligned along the patterns [12,21,22]. As a result, high-curvature geometric patterns, such as the crests of the PDMS micropatterns, exert stronger forces on the growth cone compared to patterns with features of low curvature. This implies that the contact guidance mechanism leads to the strengthening of the traction forces along the direction of the micropattern (*x* direction in Figure 1b). The growth cone detects the external geometrical cues and orients its motion in the direction that maximizes the cell–substrate interactions. This process ultimately leads to the one-dimensional axonal growth discussed in the current study. We note that in our experiments, we have not varied the mechanical, geometrical, or biochemical properties of the growth substrate. A very fruitful avenue for future research will be to investigate the effect that different types of surface adhesion molecules and/or the non-uniform distribution of these molecules on the substrate have on axonal dynamics and the formation of neural networks. This will require the measurement of the cell—substrate traction forces and the quantification of the density of cell-surface receptors using fluorescence and immunostaining techniques. 

Periodic geometrical patterns with dimensions in the range of 1–10 μm are found in vivo, functioning as physiological scaffolds for neuronal growth. These scaffolds include structures such as radial glial fibers, tracks of extracellular matrix proteins, and curved brain foldings [1,2,3,4,5,6,7]. Our results show that growth substrates with microscale periodic patterns promote the extension of the axons along the direction of the pattern. The spatial periods of the micropatterns used in our studies are relevant not only to neuronal growth within living organisms but also have great potential for applications in engineering biomimetic devices and implants aimed at nerve repair and regeneration. The nervous system possesses a remarkable ability to repair itself, but its regenerative capacity is limited following injury. Novel neuroprosthetic biomaterials serve as in vivo scaffolds for guiding regenerated axons toward their target locations, ultimately restoring connectivity and functionality. 

Our research sets the stage for further investigations of neuronal growth and, more generally, the contact guidance mechanism. The theoretical biased random walk model introduced in this paper is the simplest model that accounts for the experimental data in our simplified growth environments and predicts the cell motility coefficient and the axonal mean squared length. This model could be further generalized to include the explicit dependence of axonal growth on the cell–substrate and cell–cell interactions. This refined model could be employed to analyze the growth of other types of neurons (hippocampal, peripheral, etc.), as well as neuronal growth on nerve scaffolds, neuroprosthetic biomaterials, and in vivo growth of the nerve tissue. This approach could also be applied to study the movement of other types of cells, thus providing new insight into the nature of cellular motility. In future experiments, the use of specific fluorescent markers for neuron staining will permit the image of the structural components of the growth cone (lamellipodia, filopodia), identify the distribution of actin filaments and microtubules inside the cell, as well as directly measure traction forces during growth. These future investigations will allow us to quantify the effect of geometrical and mechanical cues on the formation of neuronal networks and to connect the observed neuronal growth behavior to internal cellular processes, such as signal transduction, cytoskeletal dynamics, and cell–surface interactions. 

## 6. Conclusions

In this paper, we have presented a combined experimental and theoretical analysis of neuronal growth on surfaces with micropatterned periodic geometrical features. We have demonstrated that the extension of axons on these surfaces is described by a biased random walk model in which the surface geometry imparts a constant drift term to the growth cone, and the random components produce a Brownian-like motion around the average growth direction. We have shown that this model is in excellent agreement with experimental data obtained for the growth cone average velocity, mean squared velocity, and axonal mean squared length. Our results indicate that the movement of the growth cone is governed by a contact-guidance mechanism arising from the cellular response to the external periodic geometry: the growth cone senses geometrical cues and aligns its motion along the surface micropatterns. The stochastic model presented in this study could be further refined to develop novel bio-inspired models for describing the formation of neural networks. The model could also be used to describe the dynamics of different cell types that respond to other environmental cues, such as electric fields, various substrate biomechanical parameters, or external biomolecules with different concentration gradients. 

## Data Availability

The data presented in this study are available within the manuscript and its Supplementary Materials.

## Supplementary Materials

The following supporting information can be downloaded at: https://www.mdpi.com/article/10.3390/biomimetics8020267/s1: Details on surface preparation and cell culture, and details on fluorescence and AFM images.
