# Supplementary material for: Biased Random Walk Model of Neuronal Dynamics on Substrates with Periodic Geometrical Patterns
Source: Biomimetics (Basel). 2023 Jun 20;8(2):267. doi: 10.3390/biomimetics8020267 (PMC10296521; doi:10.3390/biomimetics8020267)
Supplement: Supplementary file 1 [file biomimetics-08-00267-s001.zip › biomimetics-2438257-supplementary.pdf]

Surface preparation and cell culture

To fabricate micro-patterned substrates we start with 20mL polydimethylsiloxane (PDMS) solution (Silgard, Dow Corning) and pour it over diffraction gratings with slit separation of 7 μm and total surface area 25 x 25 mm^2 (Scientrific Pty. and Newport Corp. Irvine, CA). The PDMS films were left to polymerize for 48 hrs at room temperature, then peeled away from the diffraction gratings and cured at 55^0 C for 3 hrs. We use AFM imaging to ensure that the pattern was successfully transferred from the diffraction grating to the PDMS surface (Figure 1). The result is a series of periodic patterns (parallel lines with crests and troughs) with constant distance d = 7 μm between two adjacent lines. The AFM image in Figure 1(a) shows that the patterns are periodic and have constant depth. The surfaces were then glued to glass slides using silicone glue and dried for 48 hours. Next, each surface was cleaned with sterile water and spin-coated with 3 mL of Poly-D-lysine (PDL) (Sigma-Aldrich, St. Louis, MO) solution of concentration 0.1 mg/mL. The spinning was performed for 10 minutes at 1000 RPM. Prior to cell culture the surfaces have been sterilized using ultraviolet light for 30 minutes. We have performed AFM measurements and demonstrated that the topographical and mechanical properties of the PDL -coated PDMS surfaces does not change significantly across the growth substrates [14].

Cortical neurons have been obtained from rat embryos (day 18 embryos obtained from Tufts Medical School). The brain tissue protocol was approved by Tufts University Institutional Animal Care Use Committee and complies with the NIH guide for the Care and Use of Laboratory Animals. The cortices have been incubated in 5 mL of trypsin at 37ºC for 20 minutes. To inhibit the trypsin we have used 10 mL of soybean trypsin inhibitor (Life Technologies, Grand Island, NY). Next, the neuronal cells have been mechanically dissociated, centrifuged, and the supernatant was removed. After this step the neurons have been re-suspended in 20 mL of neurobasal medium

(Life Technologies, Grand Island, NY) enhanced with GlutaMAX, b27 (Life Technologies, Grand Island, NY), and pen/strep. Finally, the neurons have been re-dispersed with a pipette, counted, and plated on PDL coated glass, or PDL coated PDMS substrates, at a density of 4,000 cells/cm^2.

Fluorescence and AFM Imaging

For fluorescence imaging the cortical neurons cultured on glass or PDMS surfaces, were rinsed with phosphate buffered saline (PBS) and then incubated for 30 minutes at 37ºC with 50 nM Tubulin Tracker Green (Oregon Green 488 Taxol, bis-Acetate, Life Technologies, Grand Island, NY) in PBS. The samples were then rinsed twice with PBS and re-immersed in PBS solution for imaging. Fluorescence images were acquired using a standard Fluorescein isothiocyanate -FITC filter: excitation of 495 nm and emission 521 nm. We have previously shown that both untreated and chemically modified neurons grown in the MFP3D fluid cell remain viable over long periods of time [10-12, 16-22]. Axon outgrowth was tracked using ImageJ (National Institute of Health). All surfaces were imaged using an MFP3D Atomic Force Microscope (AFM), equipped with a BioHeater closed fluid cell, and an inverted Nikon Eclipse Ti optical microscope (Micro Video Instruments, Avon, MA). The AFM topographical images of the surfaces were obtained using the AC mode of operation, and AC 160TS cantilevers (Asylum Research, Santa Barbara, CA). Surfaces were imaged both before and after neuronal culture, and no significant change in topography was observed.
